# Heterochromatin Protein 1: A Multiplayer in Cancer Progression

**DOI:** 10.3390/cancers14030763

**Published:** 2022-02-01

**Authors:** Yu Hyun Jeon, Go Woon Kim, So Yeon Kim, Sang Ah Yi, Jung Yoo, Ji Yoon Kim, Sang Wu Lee, So Hee Kwon

**Affiliations:** 1 College of Pharmacy, Yonsei Institute of Pharmaceutical Sciences, Yonsei University, Incheon 21983, Korea; uhyun953@yonsei.ac.kr (Y.H.J.); gowoon@yonsei.ac.kr (G.W.K.); lizkimsoyeon@yonsei.ac.kr (S.Y.K.); jungy619@yonsei.ac.kr (J.Y.); jiyoon1323@yonsei.ac.kr (J.Y.K.); tkddn407@yonsei.ac.kr (S.W.L.); 2 School of Pharmacy, Sungkyunkwan University, Suwon 16419, Korea; angelna88@skku.edu

**Keywords:** HP1, cancer, DDR, epigenetic reader, chromatin dynamics

## Abstract

**Simple Summary:**

Heterochromatin protein 1 is a histone code reader protein that recognizes histone H3 lysine 9 methylation. It acts as a transcriptional corepressor by forming heterochromatin. Since its discovery, the crucial roles of heterochromatin protein 1 in tumorigenesis have been constantly reported. Indeed, numerous studies report on the altered expression level of heterochromatin protein 1 in various cancers. The changed expression pattern of heterochromatin protein 1 is associated with tumorigenesis as it regulates diverse mechanisms such as heterochromatin formation, transcriptional regulation, DNA repair, cell cycle, and telomere maintenance. Despite the studies suggesting its role in tumorigenesis, the precise tumorigenic mechanism of heterochromatin protein 1 in each cancer type is still not established. In the present review, we summarize the studies conducted on the relationship between heterochromatin protein 1 and cancer. Also, we highlight the possibility of utilizing heterochromatin protein 1 as a prognostic marker and a therapeutic target of cancer.

**Abstract:**

Dysregulation of epigenetic mechanisms as well as genomic mutations contribute to the initiation and progression of cancer. In addition to histone code writers, including histone lysine methyltransferase (KMT), and histone code erasers, including histone lysine demethylase (KDM), histone code reader proteins such as HP1 are associated with abnormal chromatin regulation in human diseases. Heterochromatin protein 1 (HP1) recognizes histone H3 lysine 9 methylation and broadly affects chromatin biology, such as heterochromatin formation and maintenance, transcriptional regulation, DNA repair, chromatin remodeling, and chromosomal segregation. Molecular functions of HP1 proteins have been extensively studied, although their exact roles in diseases require further study. Here, we comprehensively review the studies that have revealed the altered expression of HP1 and its functions in tumorigenesis. In particular, the distinctive effects of each HP1 subtype, namely HP1α, HP1β, and HP1γ, have been thoroughly explored in various cancer types. We also highlight how HP1 can serve as a potential biomarker for cancer prognosis and therapeutic target for cancer patients.

## 1. Introduction

Heterochromatin protein 1 (HP1) was first found in *Drosophila melanogaster* as a non-histone chromosomal protein that affects gene silencing via heterochromatin formation and structure maintenance [1]. HP1 families are highly conserved in most eukaryotes, ranging from yeast to humans. There are three paralogs in fission yeast (Swi6, Chp1, and Chp2), five in flies (HP1a, HP1b, HP1c, HP1d, and HP1e), and three in mice and humans (HP1α, HP1β, and HP1γ) [2]. HP1 proteins in humans, mice, and flies are made up of 173 to 240 amino acids. These HP1 proteins are smaller than those in yeasts, yet share about 50% sequence similarity. Recently, it was reported that human tissues express another HP1γ splice variant (short HP1γ, sHP1γ), which lacks the C-terminal chromoshadow domain (CSD) [3].

**Figure 1:**
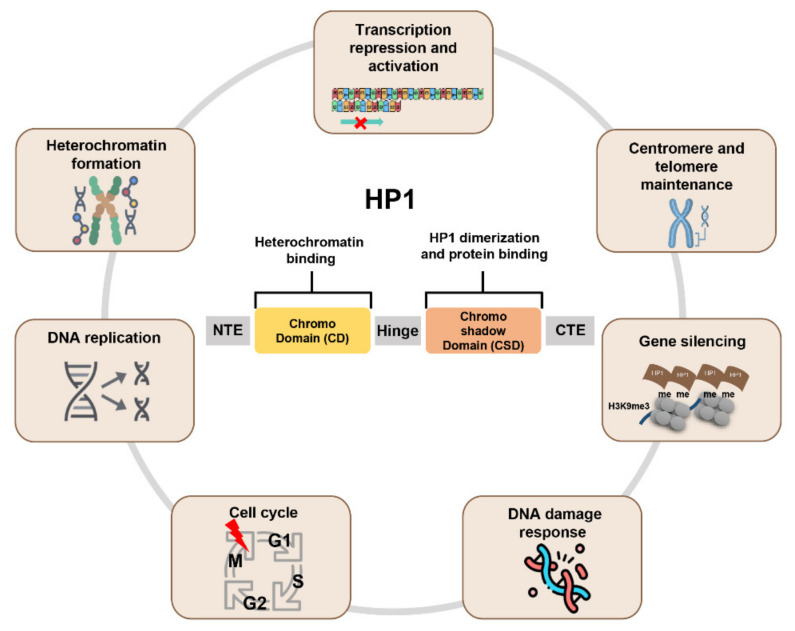
Schematic representation of HP1 function. All HP1 proteins have a chromodomain (CD) and chromoshadow domain (CSD). These domains are linked by the hinge. CD recognizes and interacts with the histone H3K9me3. CSD forms the HP1 dimer and interacts with other binding partners to perform various functions.

Information about the structure and function of HP1 has accumulated enormously. The HP1 domain consists of the chromodomain (CD) at the N-terminus and CSD at the C-terminus, connected by the hinge region [4,5] (Figure 1). In addition to the two folded domains, HP1 consists of three disordered regions—the N-terminal extension (NTE), the hinge, and the C-terminal extension (CTE) [6]. The CD domain is a specific reader that is responsible for the binding of HP1 to dimethylated and trimethylated lysine 9 of histone H3 (H3K9me2 and H3K9me3, respectively), which are hallmarks of epigenetic silencing and responsible for the recruitment of HP1 to heterochromatin [7]. The CSD domain is crucial for homo- and heterodimerization and interaction with other PXVXL-motif-containing proteins, such as chromatin modifiers, nuclear architectural proteins, and transcriptional regulators. Additionally, the PXVXL-motif-containing proteins promote the stability of CSD-CSD dimerization. CSD-CSD dimerization allows HP1 to interconnect two nucleosomes. The hinge region interacts with linker histone H1 and DNA, stabilizing HP1 chromatin binding [8]. Additionally, the hinge region interacts with CTE to form an autoinhibited and compact conformation of HP1α. Human HP1α protein exhibits a liquid-liquid phase separation pattern by forming liquid droplets upon phosphorylation in the NTE region [9]. Phosphorylated NTE interacts with the basic residues of the hinge region in HP1α, but not in HP1β and HP1γ.

Since its discovery in Drosophila and humans, HP1 has been extensively studied by numerous research groups to elucidate its biological functions. Heterochromatin formation is the most studied function of HP1. Among the chromatin structures, heterochromatin is a condensed form of the chromatin, in which transcription is inactivated or silenced. The epigenetic modification of histone H3 lysine 9 methylation (H3K9me) by SUV39H1 and SETDB1 occurs in such heterochromatin sites. The CD of HP1 recognizes this modification and recruits itself to help form a transcriptionally silenced heterochromatin [10,11]. Then, dimerization of CSD of HP1 induces the initiation and propagation of self-association, silencing euchromatin and heterochromatin formation (Figure 1).

In addition to its involvement in heterochromatin formation, HP1 is known for its role in cellular processes, including transcriptional regulation, DNA damage response (DDR), cell cycle, centromere and telomere maintenance, and splicing. Genomic instability is one of the most common characteristics of human tumors, and HP1 is closely related to DNA repair mechanisms. When DNA damage occurs, γ-H2AX and phosphorylated KRAB-associated protein-1 (KAP1) levels elevate rapidly at the damage loci [12,13]. HP1α modulates the phosphorylation of KAP1. Therefore, cells lacking HP1α fail to form pKAP1 foci after the DNA damage [14]. Additionally, HP1α is recruited to the DNA damage site by p150CAF-1 and promotes homologous recombination (HR) repair by recruiting HR components such as breast cancer type 1 (BRCA1), TP53-binding protein 1 (53BP1), and RAD5. In addition to HP1α, HP1β is also associated with DNA repair. Following the DNA damage, HP1β is released from chromatin by breaking the hydrogen bond with H3K9 methylation after phosphorylation of HP1β on threonine 51 (Thr 51) [15]. Phosphorylation of HP1β promotes γ-H2AX phosphorylation, recruiting molecules that sense the DNA breaks and activates the DNA damage response.

HP1 regulates chromatin remodeling, which is necessary for the cell cycle. HP1 proteins are released from the heterochromatin during the M phase but H3K9me3 remains intact [16]. During the M phase, Aurora kinase induces H3 phosphorylation at serine 10, which is important for the dissociation of HP1 from chromatin. HP1α and HP1β associate with centromeres and telomeres, which comprise the heterochromatic regions and modulate transcriptional gene silencing. Additionally, HP1γ is found in the telomeres in the S phase, which is required for maintaining cohesion in telomeres [17]. HP1γ interacts with the HP1-binding motif, PXVXL, in the C-terminal domain of TIN2. The HP1 binding site of TIN2 is important for sister telomere cohesion and telomere length maintenance. Numerous studies have demonstrated that abnormal expression of HP1 can promote tumorigenesis. HP1 is variably expressed in different cancer types and its altered expression is closely related to cancer proliferation, differentiation, invasion, metastasis, and tumorigenesis [18]. The altered expression of HP1 varies depending on the cancer type. Therefore, it is necessary to study the changes in the expression of HP1 in various cancers and to study the mechanisms related to gene expression. This review focuses on the altered expression and the role of HP1 in the regulation of oncogenes or tumor suppressors in cancer.

## 2. Altered Expression and Roles of HP1s in Cancer

### 2.1. Breast Cancer

Numerous studies have reported the individual role and significance of each HP1 protein in breast cancer progression [19,20,21,22,23,24]. Although HP1β and HP1γ are either up- or downregulated in different cancer types, HP1α is markedly and consistently upregulated in several types of cancers, including breast cancer [24]. The expression levels of all three subtypes of HP1 are positively correlated with that of Ki-67, a cell proliferation marker in breast cancer. Depletion of HP1α, but not the other two subtypes, causes defects in chromosome segregation and cell cycle by inducing mitotic defects [23]. Furthermore, overexpression of HP1α correlates with a prognostic value in breast cancer patients.

A study by Zhou et al. demonstrated that a histone deacetylase (HDAC) inhibitor, LBH589, resensitizes estrogen receptor (ER)-negative breast cancer cells to endocrine therapies by reactivating ER expression [25]. LBH589 reactivates ER expression by removing repressive chromatin marks and disturbing the interaction of HP1α with ER promoters in ER-negative breast cancer, preventing the formation of heterochromatin. In ERα-positive breast cancer, distant estrogen response elements (DEREs) located on chromosome 20q13 are amplified and translocated to other chromosomes [26]. Estrogen stimulation enhances scattered DERE to form depots for synchronized gene expression. DERE depots are tethered to HP1, an essential epigenetic factor in DERE-directed long-range gene repression. The dysfunction of HP1 deregulates the DERE-regulated depot formation and tumor suppressor gene transcription. Therefore, HP1α may serve as a potential prognosis marker and as a novel therapeutic target for treating both ER-positive and ER-negative breast cancer. Meanwhile, early growth response 1 (EGR1) is a stress response transcription factor whose expression is lost in breast cancer [27]. Oncogene T-box transcription factor 2 (TBX2) interacts with EGR1 and suppresses the EGR1 target gene. HP1 interacts with TBX2 and KAP1. The TBX2-KAP1-HP1 complex co-represses NDRG1 (N-Myc downstream regulated 1), which is a breast tumor suppressor. Because TBX2 is difficult to target, these functional multicomponent complexes, including HP1, could be alternative therapeutic targets for TBX2-expressing breast cancers.

While some studies show that HP1α promotes the cell proliferation of breast cancer cells, some studies report that HP1α suppresses cell migration and invasion [28]. HP1α is decreased in invasive breast cancer cells, i.e., MDA-MB-231 and HS578T, compared to non-invasive breast cancer cells, i.e., MCF-7 and T47D. Additionally, the overexpression of HP1α decreases the invasive potential of MDA-MB-231 cells. Consistent with the in vitro results, tumor tissues from metastatic lesions showed lower HP1α expression compared to the tumor tissues from nonmetastatic lesions [29]. Another study demonstrated that transcription factor yin yang 1 (YY1) has an important role in the differential transcriptional activity of the HP1α [22]. YY1 and HP1α are both downregulated in invasive breast cancer cells and YY1 knockdown decreases the mRNA level of HP1α. The overexpression of YY1 in HS578T cells downregulates cell migration independent of HP1α. These results show that YY1 positively regulates HP1α by binding to the HP1α gene promoter in non-invasive breast cancer cells but not in invasive breast cancer cells.

HP1 family proteins are known to affect the DDR pathway and induce tumorigenesis [21]. HP1 promotes tumorigenesis by impairing the tumor-suppressing function of BRCA1. HP1 knockdown causes genotoxic stress and delays the DNA repair system by reducing the recruitment of BRCA1. Additionally, HP1 depletion dysregulates the formation of both BRCA1 and 53BP1 foci, leading to defective double-strand break (DSB) repair and increasing apoptosis after irradiation. The same group further reported the function of HP1β in regulating the DDR pathway in breast cancers [23]. About 60% of breast cancer patients show high HP1β expression, while the rest of them show no or low expression of each HP1 protein. An upregulated HP1β mRNA level also correlates with poor differentiation of breast tumors and lower survival rates in breast cancer patients. HP1β depletion in breast cancer cells largely increases the sensitivity to poly(ADP-ribose)polymerase (PARP) inhibitor, which suppresses DNA repair. MCF7 cells with wild-type BRCA1 are resistant to PARP inhibitor treatment. However, HP1β knockout MCF7 cells become sensitive to the PARP inhibitor (ABT-888). This implies that HP1β can be a predictive biomarker for synthetic lethality with PARP inhibitor, as well as a prognostic marker for breast cancer.

In addition to HP1α and HP1β, HP1γ has unique functions in tumorigenesis [18]. Although HP1γ expression is variable depending on the tumor type, HP1γ expression is maintained in triple-negative breast cancer (TNBC) [30]. HP1γ binds to the LxVxL motif of lysine demethylase 2A (KDM2A) and controls ribosomal RNA (rRNA) transcription by stimulating the nuclear accumulation of KDM2A. Knockdown of HP1γ in TNBC cells reduces KDM2A accumulation in the nucleolus and represses the reductions in rRNA transcription and cell proliferation under glucose starvation. Therefore, the capability of HP1γ to regulate rRNA transcription could be possibly utilized for the treatment of TNBC.

### 2.2. Ovarian Cancer

HP1 appears to play a significant role in the progression of ovarian cancer. 17-Allylamino-17-demethoxygeldanamycin (17AAG), an inhibitor of heat shock protein 90 (HSP90), has an anticancer effect by inhibiting the degradation of multiple client oncogenic proteins [31]. The treatment of ovarian cancer cells with 17AAG changes the expression of chromatin-associated proteins. Furthermore, 17AAG increases HP1γ, although it decreases histone acetyltransferase 1 and histone arginine methyltransferase. Moreover, another study revealed that HP1γ was upregulated in chemotherapy-sensitive ovarian cancers, while it was lowered in chemotherapy-resistant ovarian cancers [32]. Although the precise mechanism is unknown, these studies suggest that HP1γ has an anticancer role in ovarian cancer.

HP1 can regulate the response to chemotherapy in ovarian cancer by modulating DDR. Checkpoint kinase 1 inhibitor LY2606368, which is currently in a clinical trial for high-grade serous ovarian cancer (HGSOC) patients, impairs DNA damage response [33]. The suppression of bromodomain-containing protein 4 (BRD4), which is overexpressed in HGSOC, inhibits HP1α, enhancing the cytotoxicity of LY2606368. BRD4 directly interacts with the HP1α promoter and downregulates HP1α expression to facilitate DDR. Aside from BRD4, another epigenetic regulator, HDAC2, is involved in increased HP1 expression in cisplatin-treated PEO1 ovarian cancer cells [34]. Similarly, HDAC2, HP1α, and HP1β expression are increased after carboplatin treatment in OV1002, a carboplatin-sensitive xenograft, but not in HOX424, a carboplatin-insensitive xenograft. Additionally, HDAC2 knockdown reduces HP1 expression and induces DSB. Based on these studies, the altered level of HP1 expression could be a prognosis marker and chemotherapy response marker in ovarian cancer.

### 2.3. Cervical Cancer

The main risk factor in cervical cancer is high-risk human papillomavirus (HPV) infection. High-risk HPV oncoprotein E6 induces abnormal nuclear export of HP1γ but not HP1α and HP1β during cervical cancer progression [35]. Additionally, HPV-infected patients show high expression levels of HP1γ. HPV mediates the nuclear export of human HP1γ and leads to p53 instability by enhancing UBE2L3-mediated polyubiquitination on p53. Indeed, the suppression of the nuclear export of HP1γ rescues the p53 level and reduces the tumorigenesis of cervical cancer. These findings show that both the expression level and cellular localization of HP1γ play crucial roles in the tumorigenesis of cervical cancer. As a result, the inhibition of abnormal cytoplasmic localization of HP1γ could be a potential therapeutic strategy for cervical cancer patients.

Cullin4B (CUL4B) is an E3 ubiquitin ligase that is associated with tumorigenesis and is overexpressed in human cervical cancer [36]. HP1 forms a complex with CUL4B, SUV39H1, and DNA methyltransferase 3A (DNMT3A). This complex reduces a collection of genes, including insulin-like growth factor-binding protein 3 (IGFBP3), a tumor suppressor, by regulating gene transcription. This study suggests that CUL4B enhances the proliferation and invasion of cervical cancer by repressing its target genes. Additionally, it shows that HP1 can affect tumorigenesis by coordinating with other epigenetic regulators.

The poor prognosis of cervical cancer can be attributed to the high rate of metastasis [37]. Matrix metalloproteinases (MMPs) are important factors in tumor progression and metastasis. MMP3 upregulates heat shock protein (HSP) family members by cooperating with heat shock transcription factor 1 (HSF1) [38]. The hemopexin-like repeat (PEX) domain of MMP3 is essential for HSP70B’ gene transcription. HP1α and HP1γ cooperate with the PEX domain for the activation of HSP70B mRNA. Since HSP proteins are elevated in cervical cancer cells and activated HSF1 is associated with a poor prognosis of cancer, HP1 may be a crucial factor contributing to this process. Almost 10% of cervical cancers use recombination-mediated alternative lengthening of telomere (ALT) to avoid telomere shortening [39]. Cells using ALT are characterized by ALT-associated PML nuclear bodies (APBs). APBs, induced by p53/p21, are related to cell growth arrest and senescence. HP1α and HP1γ mediate APB formation, inducing senescence. Taken together, HP1 proteins play an important role in cervical cancer tumorigenesis and cell senescence.

### 2.4. Colorectal Cancer (CRC)

Three HP1 proteins are overexpressed in CRC tissues compared to normal tissues [40]. Overexpression of HP1α is correlated with the short overall survival (OS) rate of CRC patients. Additionally, high expression of HP1γ is associated with colon cancer progression and reduces disease-free survival rates [40]. HP1γ promotes cancer cell proliferation and cell cycle progression in CRC by downregulating the expression of cyclin-dependent kinase 6 (CDK6) and p21. Moreover, miR-30a, a tumor-suppressive microRNA, targets HP1γ and inhibits tumor growth [41]. Taken together, the miR-30a-HP1γ-p21 regulatory axis plays an important role in CRC development and may be utilized as a prognostic marker and a therapeutic target for cancer growth inhibition. Conversely, a previous study reported that HP1 is associated with the suppression of tumorigenesis [42]. A proapoptotic tumor suppressor, KRAB-zinc finger transcription factor ZNF382, interacts with HP1β and suppresses tumorigenesis by modulating heterochromatin formation.

Several studies have shown that HP1β is negatively associated with metastasis and invasion of CRC. The downregulation of HP1β in several CRC cell lines increases the metastasis of cancer by upregulating the mRNA level of MMP2 and membrane type 1 metallopeptidase (MT1-MMP) [43]. Furthermore, the expression of HP1α is enhanced in Caco-2 and HT-29 cells, which express aberrantly high levels of gastrin-releasing peptide (GRP) and its receptor (GRPR). Upregulation of GRP and GRPR in CRC cells attenuates metastasis and increases patients' survival rate [44]. Inhibition of GRP/GRPR signaling significantly downregulates the expression of HP1β and increases the invasion of Caco-2 cells [45].

The expression level and localization of HP1 can be modified by epigenetic enzymes. HDAC inhibitor decreases the expression of three HP1 proteins in HT29 cells [46]. HDAC inhibitor induces hyperacetylated environments of centromeres and repositions HP1 proteins into the internal part of the nucleus. After treating trichostatin A (TSA), which is a pan-HDAC inhibitor, HP1 relocates to the centromeric chromatin in CRC HCT116 cells but not in non-tumoral cells [47]. This difference is associated with chromosome instability caused by TSA-induced hyperacetylation and reduced H3K9me3.

### 2.5. Lung Cancer

Similar to CRC, HP1 proteins are also overexpressed in lung cancer. Lung cancer is categorized into small cell lung cancer (SCLC) and non-small cell lung cancer (NSCLC). NSCLC can be classified into lung adenocarcinoma (LUAD) and lung squamous cell carcinoma (LUSC). The expression level of HP1α is significantly increased in tissues and metastatic lesions of LUAD patients compared to their normal adjacent tissues (n = 20) [48]. LUAD patients with high HP1α expression have worse OS. Furthermore, a xenograft mouse model of lung CD133+ tumor stem-like cells with HP1α knockdown showed a significant reduction in tumor volume and decreased level of metastatic foci. A few studies have investigated the molecular mechanism of HP1 in lung cancer progression. Since HP1 regulates the DDR pathway [5,12,14,15], its dysregulation would disrupt proper DNA repair, leading to lung cancer progression. For instance, RAD6, an E2 ubiquitin-conjugating enzyme, regulates HP1α and performs HR in DNA DSB repair [49,50,51]. RAD6 ubiquitinates HP1α at K154 and boosts HP1α degradation, thereby forming an open chromatin structure and promoting HR. The levels of RAD6 and HP1α are negatively correlated, and NSCLC patients with low RAD6 and high HP1α expression have low survival rates. Despite the number of studies indicating the tumorigenic role of HP1α in lung cancer, the exact function of HP1α in lung cancer remains controversial. Dutta et al. revealed that unphosphorylated STAT3 interacts with HP1α and promotes heterochromatin formation, thereby suppressing cell proliferation and tumor growth in lung cancer [52].

In addition to HP1α, the expression of HP1β and HP1γ correlates with poor clinical outcomes in patients with LUAD [53,54]. Out of the three HP1 proteins, HP1γ is the most amplified histone reader protein in LUAD. The mRNA level of HP1γ is correlated with tumor size, lymph node metastasis, and poor outcome in LUAD patients. Inhibition of HP1γ in vivo reduces tumor size and extends the survival of KRASG12D-induced LUAD mice [54]. Mutation or depletion of HP1γ significantly decreases the proliferation and migration ability of LUAD cells. HP1γ positively regulates the expression of oncogenes including *ELK1, AXL*, and *PVT* by downregulating the expression levels of *NCOR2* and *ZBTB7A*, the transcription-repressive regulators. High HP1γ levels are negatively associated with *NCOR2* and *ZBTB7A* levels, so LUAD patients with high levels of HP1γ show poor prognosis. KEGG pathway enrichment analysis in LUAD patient tissue demonstrated that HP1γ is involved in regulation of the cell cycle and the p53 signaling pathway [53]. HP1γ is significantly upregulated in NSCLC, and the high prevalence of HP1γ is also associated with EGFR mutation (40 out of 42 patients) [55]. These results demonstrate that HP1γ may serve as a prognostic marker and promising therapeutic target for lung cancer treatment.

### 2.6. Liver Cancer

HP1β and HP1γ are highly expressed in hepatocellular carcinoma (HCC) compared to normal tissue. Abnormal expression levels of the two proteins highly correlate with unfavorable outcomes for HCC patients [56,57]. A high expression level of HP1β promotes cancer cell proliferation and migration in vitro [57]. HP1β activates the Wnt/β-catenin pathway by interacting with a transcription factor, high mobility group AT-hook 2 (HMGA2). The suppression of the Wnt/β-catenin pathway by β-catenin siRNA or its inhibitor reduces HP1β-mediated cell growth. Likewise, overexpression of HP1γ enhances the proliferation of HCC cells and causes reduced OS and recurrence-free survival [56]. Thus, HP1β and HP1γ could be considered as potential prognostic biomarkers in HCC. Aside from HP1β and HP1γ, HP1α is also associated with tumor cell proliferation and viability by modulating liver cell metabolism. Inactivation of HP1α in HepG2 cells modulates the s-adenosyl methionine (SAM) pathway by suppressing the transcription of methionine adenosyltransferase 2A (*MAT2A*) which is a crucial gene in the SAM pathway. HP1α regulates *MAT2A* expression by reducing the recruitment of H1β and HP1γ to *MAT2A* promoters.

HP1 proteins are also involved in hepatocarcinogenesis by regulating telomerase activity [58]. Toll-like receptor 4 (TLR4), one of the inflammatory factor receptors, enhances the expression of histone lysine 9 methyltransferase SUV39H2 and promotes the formation of the H3K9me3-HP1-telomeric repeat binding factor 2 (TRF2) complex, which maintains the length of the telomere. TLR4 promotes the interaction between HP1α and DNMT3b, inhibiting telomeric repeat-containing RNA (TERRA) transcription and ultimately enhancing the telomerase activity and carcinogenesis. The carcinogenic action of TLR4 requires triple complexes of the three HP1 proteins (HP1α, HP1β, and HP1γ). In addition to TLR4, double-mutant p53 (N340Q/L344R) also promotes hepatocarcinogenesis by activating telomerase [59]. Double-mutant p53 binds to the pyruvate kinase M2 (PKM2) promoter and enhances its expression, and also increases phosphorylation of histone H3 threonine 11 (pH3T11). Furthermore, pH3T11 increases H3K9me1, and this modification works with HP1α to upregulate Pim1 kinase, which increases telomere reverse transcriptase and reduces TERRA expression. Ultimately, p53 (N340Q/L344R) activates telomerase and maintains telomere extension, thereby contributing to liver cancer progression.

### 2.7. Pancreatic Cancer

Overexpression of HP1γ is associated with reduced OS and disease-free survival in pancreatic ductal adenocarcinoma (PDAC) patients. HP1γ enhances cell proliferation, migration, invasion, and anchorage-free growth of PDAC [60]. HP1γ facilitates PDAC by regulating cell cycle transition with increased CDK1 and proliferating cell nuclear antigen (PCNA). In addition to regulating CDK, HP1 modulates CDK inhibitors such as p15INK4b. The nuclear factor of activated T cells (NFAT) c2, an oncogenic transcription factor, enhances pancreatic cancer growth by suppressing p15INK4b, a tumor suppressor. NFATc2 binding to the p15INK4b promoter recruits SUV39H1 and HP1γ to the NFATc2 repressor complex. Such recruitment induces facultative heterochromatin formation and p15INK4b gene silencing [61]. In addition to its role in cell cycle, HP1γ regulates aerobic glycolysis by modulating the expression of fructose bisphosphatase 1 (FBP1). HP1γ inhibits the expression of FBP1, a negative regulator of aerobic glycolysis [62]. Collectively, HP1γ promotes pancreatic cancer progression by regulating cell cycle and glycolysis and may serve as a potential therapeutic target for pancreatic cancer.

Although most studies highlight the oncogenic role of HP1, a few studies have specifically reported on the tumor-suppressing role of HP1 in pancreatic cancer. Lomberk et al. revealed that Krüppel-like factor 11 (KLF11), a transcription factor that mediates tumor suppression, recruits HP1α to its target gene promoters by interacting with the PXVXL domain in KLF11 [63]. Furthermore, recruitment of HP1α and SUV39H1 by KLF11 suppresses the activation of genes regulated by KLF11, which consequently promotes apoptosis and inhibits pancreatic cancer growth.

### 2.8. Prostate Cancer

Androgen receptor (AR), a ligand-dependent transcription activator, is crucial in prostate cancer development and tumor malignancy [64,65]. Androgen signaling contributes to the development and proliferation of prostate cancer [66]. In prostate cancer, the expressions levels of HP1β correlate with a higher Gleason score, the most common grading system of prostate cancer. As a cofactor of AR, HP1β upregulates the transcription of AR target genes and contributes to cancer proliferation. HP1β is overexpressed in castration-resistant prostate cancer (CRPC), which is an androgen depletion therapy-resistant prostate cancer. Silencing of HP1β abolishes prostate cancer growth. In addition to that of HP1β, knockdown of HP1γ downregulates AR expression and the proliferation of prostate cancer [67]. On the other hand, another study showed that HP1γ plays an AR-independent role in CRPC progression. For example, HP1γ and BRD4 co-occupy in the genome and cooperate to modify the transcription of their shared target genes, such as *CCNB1, MTBP*, and *SASS6* [49]. The depletion of either BRD4 or HP1γ inhibits these target genes, which could be a novel strategy in targeting CRPC in an AR-independent manner. Additionally, HP1γ is significantly overexpressed in localized and metastatic lesions of prostate cancer, and elevation of HP1γ correlates with poor clinical outcomes [68]. Additionally, c-Myc takes part in HP1γ upregulation by binding to the first intron of the HP1γ gene, while HP1γ/miR-451a/c-Myc plays an important role in prostate cancer progression [69]. Collectively, these results suggest that HP1β and HP1γ stand as promising therapeutic targets in prostate cancer.

### 2.9. Osteosarcoma

HP1γ overexpression in human sarcoma tissues may lead to diseases such as osteosarcoma. High expression levels of HP1γ are correlated with poor prognosis in osteosarcoma. Inhibition of HP1γ induces downregulation of cell growth, increased apoptosis, and cell cycle arrest in the G0/G1 phase [70]. In addition, HP1 is involved in the expression of the SET nuclear proto-oncogene (SET), which modulates DDR and repairs the chromatin surrounding DSBs [71]. In U2OS cells, deletion of SET increases DDR and cell survival after treatment with radiomimetic drugs, whereas overexpression of SET inhibits HR-mediated DNA repair. SET interacts with KAP1 and recruits HP1γ on chromatin, creating a repressive microenvironment for HR. Moreover, HP1 is associated with the tumor-suppressive effect of retinoblastoma protein (pRb) [72]. Additionally, pRb is methylated by Set7/9 on the c-terminal region, and methylated pRB interacts with HP1; pRB is co-expressed with HP1 and induces G1 arrest. Based on these findings, HP1 regulates DDR and the cell cycle of osteosarcoma cells.

Tumor stem cell (TSC) is the main risk factor of tumorigenesis, chemoresistance, and the malignant development of various tumors, including osteosarcoma. Saini et al. characterized the TSCs of osteosarcoma by using transcriptome, proteome, and bioinformatics analyses [73]. These analyses showed that HP1γ expression is consistently increased in TSC-enriched osteosarcoma and lung tumors metastasized from osteosarcoma. Therefore, HP1γ can be both a prognostic marker and a biomarker of cancer metastasis.

### 2.10. Glioma

HP1γ is upregulated in human glioma tissues, and overexpression of HP1γ can be used to predict dismal recurrence-free survival and poor OS [74]. HP1γ knockdown suppresses cell proliferation and the colony-forming ability of U87 cells by inducing cell cycle arrest at the G0/G1 phase and apoptosis. Additionally, HP1γ knockdown significantly increases the mRNA and protein expression of *CDKN1A*. Consistent with in vitro assays, in vivo studies on HP1γ-depleted xenografts showed decreased tumor growth and Ki-67 expression levels. The level of *CDKN1A* expression was increased in HP1γ-depleted xenograft, and cell proliferation was partially rescued via *CDKN1A* knockdown in vitro. This study indicated that HP1γ stimulates the growth of glioma cells by targeting *CDKN1A*.

### 2.11. Leukemia

Interestingly, the expression of HP1 proteins is relatively low in immune cells compared to most other types of cells [75]. The three HP1 proteins and H3K9 methylation are not found in eosinophil and neutrophil granulocytes. However, increased levels of all three HP1 proteins and H3K9 methylation are found in the granulocytes of acute myeloid leukemia (AML) and chronic myeloid leukemia (CML) patients [76]. In myeloid leukemia, incompletely differentiated immature cells accumulate, maintaining low expression levels of HP1β and HP1γ. Additionally, HP1 proteins are significantly elevated in accelerated phase and blast crisis patients compared to CML patients. During blood cell differentiation, chromatin becomes condensed and levels of epigenetic proteins change, including those of HP1. HP1 is decreased and the level of HP1 conversely correlates with the level of monocyte and neutrophil elastase inhibitor during cell differentiation of U937 cells induced by retinoic acid. This immaturity of myeloid leukemia cells is accompanied by increased DDR, allowing incompletely differentiated AML leukemic cells to respond to DSB induction [75]. Therefore, HP1 proteins could be an indicator of granulocyte maturation in AML patients. Moreover, HP1γ expression is associated with resistance to the DNA methyltransferase inhibitor, 5-azacytidine (AZA) [77]. HP1γ is decreased after AZA treatment in AZA-sensitive leukemia cells, whereas all three HP1 proteins remain unaltered in AZA-resistant leukemia cells. Knockdown of HP1γ induces apoptosis and reduces the viability of AZA-resistant leukemia cells, accompanied by downregulation of ATM/BRCA1 signaling. Therefore, the growth inhibition of AZA-resistant cells by targeting HP1γ may be caused by suppressing the DDR pathway. Based on these findings, HP1γ may be used to treat leukemia patients with AZA resistance.

### 2.12. Other Cancers

In the various cancers described above, the altered expression of HP1 is related to carcinogenesis. Although the mechanism of HP1 in tumorigenesis has not yet been elucidated, alteration of HP1 expression has been reported in many cancers. The mRNA levels of all three HP1 subtypes are highly expressed in gastric cancer (GC) [78]. Above all, HP1γ is significantly associated with poor OS, progression-free survival, and post-progression survival of GC patients. Inhibition of HP1γ significantly decreases the malignant phenotype of GC. RNA-Seq analysis demonstrates that HP1γ is related to the cell cycle, mismatch repair, and interferon signaling pathway in GC [79]. In melanoma, it is reported that the reduced HP1β expression level correlates with the increased invasive potential of melanoma cells [80]. Similarly, loss of HP1β and also HP1α contributes to increased tumor progression and metastatic potential in thyroid cancer [81]. These findings indicate that the expression levels of HP1 and their functions vary depending on the cancer type; thus, further studies on the distinctive effects of HP1 in various cancer types should be explored.

## 3. Conclusions

The molecular function of HP1 protein has been studied for a long time, and today many researchers have much interest in the physiological significance of HP1, which can be further associated with the disease phenotype. Recent papers observing the relationship between HP1 and tumorigenesis have implied that HP1 is deeply associated with tumorigenesis via modulating diverse molecular mechanisms such as cell cycle, DNA repair, and transcriptional regulation [18]. Existing review articles on the role of HP1 in cancer published several years ago [18,82] focus on the general functions of HP1 proteins rather than the individual HP1 subtype in cancer. Additionally, the cancer types studied by the researchers have greatly expanded since then. In the present study, we have reviewed the function of each HP1 subtype in various cancer types. The altered expression of HP1, which differs across diverse types of cancer (Table 1), highlights the potential of HP1 as a novel biomarker for cancer diagnosis. However, merely abnormal expression patterns of HP1 have been reported in several tumor types, such as glioma and leukemia. Accurate molecular mechanisms of HP1 contributing to tumor progression are yet to be elucidated. Thus, clarifying the molecular mechanism of the oncogenic or tumor-suppressive function of HP1 in those cancers would shed light on the development of a therapeutic strategy that is specifically effective in each cancer type.

Recently, it has been discovered that epigenetic dysregulation is crucial for tumorigenesis [83]. As one of the epigenetic regulators, HP1 induces chromatin remodeling and affects gene silencing or gene activation. However, the gene-regulatory function of HP1 has hardly been linked to carcinogenesis for decades. Future works combining transcriptomics and clinical analysis would provide more information about the entire cascade of HP1-mediated tumor progression related to misregulation of specific gene sets. Despite the increasing number of epigenetic drugs, chemicals targeting HP1 or HP1-related mechanisms in cancer have not yet been developed [15]. Hence, it is necessary to develop HP1 inhibitors and conduct further studies to selectively target each HP1 protein in cancer tissues. Lately, the role of HP1α in liquid droplet (LD) formation has been reported [9]. LDs are currently recognized as important organelles that participate in cellular functions, such as energy metabolism, cell signaling, and inflammation [84]. Moreover, LDs accumulate in neoplastic processes, and their accumulation in cancer cells is coupled to increased cancer cell proliferation, cancer aggressiveness, and resistance to death. These findings draw attention to the potential oncogenic role of HP1α by regulating LD formation. Therefore, investigating the unique function of HP1α on LD formation would be invaluable in understanding cancer physiology.

Collectively, in addition to the importance and detailed function of HP1 in cancer progression, multiple approaches utilizing HP1 as a diagnostic or predictive biomarker or therapeutic target will enable us to combat our long-fought enemy, cancer.

## Figures and Tables

**Table 1:** Alterations of HP1 expression in cancers.

Cancer Type	HP1 Subtype	Altered Expression in Cancer	Role in Cancer
Breast cancer	HP1α	Increased	Promotes tumorigenesis
		Decreased	Suppresses metastasis
	HP1β	Increased	Promotes tumorigenesis
	HP1γ	-	Controversial
Ovarian cancer	HP1α	-	-
	HP1β	-	-
	HP1γ	-	-
Cervical cancer	HP1α	-	-
	HP1β	-	-
	HP1γ	Increased	Promotes tumorigenesis
Colorectal cancer	HP1α	Increased	Promotes tumorigenesis
	HP1β	Increased	Promotes tumorigenesis
		Decreased	Suppresses metastasis
	HP1γ	Increased	Promotes tumorigenesis
Lung cancer	HP1α	Increased	Promotes tumorigenesis
	HP1β	Increased	Promotes tumorigenesis
	HP1γ	Increased	Promotes tumorigenesis
Liver cancer	HP1α	-	-
	HP1β	Increased	Promotes tumorigenesis
	HP1γ	Increased	Promotes tumorigenesis
Pancreatic cancer	HP1α	-	-
	HP1β	-	-
	HP1γ	Increased	Promotes tumorigenesis
Prostate cancer	HP1α	-	-
	HP1β	Increased	Promotes tumorigenesis
	HP1γ	Increased	Promotes tumorigenesis
Osteosarcoma	HP1α	-	-
	HP1β	-	-
	HP1γ	Increased	Promotes tumorigenesis
Glioma	HP1α	-	-
	HP1β	-	-
	HP1γ	Increased	Promotes tumorigenesis
Leukemia	HP1α	Increased	Promotes tumorigenesis
	HP1β	Increased	Promotes tumorigenesis
	HP1γ	Increased	Promotes tumorigenesis
Gastric cancer	HP1α	-	-
	HP1β	-	-
	HP1γ	Increased	Promotes tumorigenesis
Multiple myeloid	HP1α	-	-
	HP1β	-	-
	HP1γ	-	-
Melanoma	HP1α	-	-
	HP1β	Decreased	Suppresses metastasis
	HP1γ	-	-
Thyroid cancer	HP1α	-	-
	HP1β	Decreased	Promotes tumorigenesis/Suppresses metastasis
	HP1γ	Decreased	Promotes tumorigenesis/Suppresses metastasis

The dashed lines (-) mean that there is no paper reporting the altered expression of HP1 or its function in cancer.
